# Natural Antioxidant and Anti-Inflammatory Compounds in Foodstuff or Medicinal Herbs Inducing Heme Oxygenase-1 Expression

**DOI:** 10.3390/antiox9121191

**Published:** 2020-11-27

**Authors:** Dongyup Hahn, Seung Ho Shin, Jong-Sup Bae

**Affiliations:** 1 School of Food Science and Biotechnology, College of Agriculture and Life Sciences, Kyungpook National University, Daegu 41566, Korea; dohahn@knu.ac.kr; 2 Department of Integrative Biology, Kyungpook National University, Daegu 41566, Korea; 3 Department of Food and Nutrition, Institute of Agriculture and Life Science, Gyeongsang National University, Jinju 52828, Korea; shshin@gnu.ac.kr; 4 College of Pharmacy, CMRI, Research Institute of Pharmaceutical Sciences, BK21 Plus KNU Multi-Omics based Creative Drug Research Team, Kyungpook National University, Daegu 41566, Korea

**Keywords:** heme oxygenase-1, antioxidant, anti-inflammatory compound, foodstuff, medicinal herbs

## Abstract

Heme oxygenase-1 (HO-1) is an inducible antioxidant enzyme that catalyzes heme group degradation. Decreased level of HO-1 is correlated with disease progression, and HO-1 induction suppresses development of metabolic and neurological disorders. Natural compounds with antioxidant activities have emerged as a rich source of HO-1 inducers with marginal toxicity. Here we discuss the therapeutic role of HO-1 in obesity, hypertension, atherosclerosis, Parkinson’s disease and hepatic fibrosis, and present important signaling pathway components that lead to HO-1 expression. We provide an updated, comprehensive list of natural HO-1 inducers in foodstuff and medicinal herbs categorized by their chemical structures. Based on the continued research in HO-1 signaling pathways and rapid development of their natural inducers, HO-1 may serve as a preventive and therapeutic target for metabolic and neurological disorders.

## 1. Introduction

Since the discovery of heme oxygenase-1 (HO-1) by Tenhunen et al. in 1968 [1], three HO isoenzymes (HO-1, HO-2 and HO-3) have been reported in mammals [2]. Heme oxygenase (HO) mostly exists in two forms, HO-1, the inducible form, and HO-2, the constitutive form [3]. HO-3 is a pseudogene derived from the HO-2 transcript, and has any intron [4]. HO-1 and HO-2 degrade heme in an identical stereospecific manner to biliverdin with the concurrent release of carbon monoxide (CO) and iron [5]. Among those three proteins, only HO-1 was shown to be inducible by a variety of stimuli such as oxidative stress [6,7]. On the other hand, the constitutive nature of HO-2 made it less attractive as a drug target compared to HO-1.

Recent studies utilizing gene knockdown techniques and small molecule inhibitors have shown that HO-1 induction suppresses development of metabolic disorders and nerve system disorders including obesity [8], hypertension [9], atherosclerosis [10], Parkinson’s disease [11] and hepatic fibrosis [12]. A significant body of literature has focused on the mechanism of heme degradation by HO-1 and there is strong evidence that inducing HO-1 expression is an effective method of suppressing oxidative dysregulation, inadequate immune response and related disorders [13].

Chemoprevention is defined as a pharmacological approach used to suppress or reverse the disease progression [14]. Antioxidants in fruits and vegetables, such as polyphenols, have been proposed as primary chemopreventive agents [15]. Many of the natural compounds have shown the ability to induce HO-1 without cytotoxic effects. Although several reviews have dealt with natural HO-1 inducers [16,17,18], none of them categorized and visualized their chemical structures of the up-to-date list of HO-1 inducers. Given the importance of chemical structures of natural compounds on their antioxidant ability, it is necessary to provide the chemical structure-based classification of HO-1 inducers. Here, we provide a compilation of knowledge on natural antioxidants and anti-inflammatory compounds in foodstuff or medicinal herbs focusing on the diseases in which HO-1 induction could exert preventive or therapeutic effects by modulating various signaling pathways.

## 2. Beneficial Effects of Heme Oxygenase-1 Induction as Preventive Measures against Diseases

HO-1 is involved in a wide range of diseases in many types of mammalian tissue including the liver, artery and neuron. Although the effect of HO-1 induction is controversial in some types of diseases, there are several cases showing the benefits of antioxidant and anti-inflammatory effects of HO-1. Here we introduced five types of diseases in which HO-1 induction is confirmed to be beneficial by genetic knockout experiments in vitro and in vivo.

### 2.1. Obesity

Obesity, characterized by increased adipose tissue mass that results from both increased number and size of fat cells [19], is one of the major metabolic disorders worldwide [20]. Obesity is also a major risk factor in vascular dysfunction and insulin resistance that leads to hypertension and diabetes, respectively [21,22]. Decreased level of HO-1 was correlated with the inflammatory cytokine increase and insulin resistance [8,23]. In animal studies, the beneficial effect of HO-1 induction against adipocyte morphology was observed in the *ob/ob* mouse model [8] and Zucker diabetic rats [24]. Administration of 3 mg/kg of cobalt protoporphyrin (CoPP), the HO-1 inducer that causes a sustained increase in the HO-1 protein level, prevented weight gain by decreasing visceral and subcutaneous fat content compared with the vehicle group [8]. Upregulation of HO-1 attenuated adipogenesis in bone marrow by increasing serum adiponectin, and decreasing plasma tumor necrosis factor-alpha (TNF-alpha), interleukin (IL)-6 and IL-1β levels [8]. Upregulating the HO-system with hemin also reduced perirenal adiposity in the Zucker rat model by inhibiting several proinflammatory/oxidative mediators in perirenal fat including macrophage-inflammatory-protein-1α (MIP-1α), endothelin (ET-1), 8-isoprostane, TNF-α, IL-6 and IL-1β [24]. The increase of HO-1 in perirenal fat was confirmed by enzyme-linked immunosorbent assay (ELISA) showing the involvement of the obesity regulator in vivo [24]. These results indicate that the HO-1 level is negatively correlated with obesity-related symptoms and HO-1 induction ameliorates genetically induced obesity in vivo.

### 2.2. Hypertension

Hypertension, a long-term medical condition where the blood pressure in the artery is persistently elevated, is now considered as a chronic progressive disease that develops over many years [25]. Hypertension is a major risk factor of stroke, myocardial infarction, left ventricular hypertrophy and renal disease [26,27]. There are various reports showing the protective role of HO-1 in the development and progression of pulmonary arterial hypertension. In an animal study, transgenic mice that overexpress HO-1 in the lung showed reduced incidence of pulmonary hypertension and vessel wall hypertrophy induced by hypoxia [9]. The levels of proinflammatory cytokines and chemokines induced by hypoxia were also suppressed in HO-1 transgenic mice [9]. Another in vivo study using a HO-1 inducer also showed that hemin treatment abrogated the induction of pulmonary hypertension and pulmonary arterial wall thickening in rats injected with monocrotaline [28]. Adipose tissue-specific induction of HO-1 also demonstrated the beneficial effect of HO-1 against obesity-related hypertension. Induction of HO-1 lowered blood pressure levels in obese mice similar to that of lean mice [29]. These studies highlight the protective role of the HO-1 signaling in hypertensive models in vivo.

### 2.3. Atherosclerosis

Atherosclerosis is characterized by the accumulation of lipids and fibrous elements in the large arteries [30]. Since atherosclerosis was regarded as a chronic inflammatory state, the effect of HO-1 modulation in the disease has been studied extensively. Case reports on deficiency of *HMOX1*, a human gene encoding HO-1 enzyme, showed vascular injury and early atherosclerotic changes along with inflammation and nephropathy, suggesting the importance of HO-1 in vascular health [31,32,33]. The protective role of HO-1 induction against atherosclerosis was further supported by various animal studies. HO-1 overexpression by adenovirus-mediated gene transfer successfully inhibited atherogenesis in a hypercholesterolemic animal model [10]. Induction of HO-1 by pharmacological inducers also attenuated the development of atherosclerotic lesions in vivo [34,35]. On the other hand, Hmox1^-/-^ mice reportedly had growth retardation, anemia, iron deposition [36,37] and developed severe aortitis and coronary arteritis with mononuclear cellular infiltration and fatty streak formation [38]. In sum, these results found in mouse and human show that HO-1 plays a protective role against the progression of atherosclerosis.

### 2.4. Parkinson’s Disease

Parkinson’s disease is one of the major neurodegenerative disorders of uncertain pathogenesis characterized by the loss of the dopaminergic neurons [39]. The deregulation of the HO system has been associated with many types of neurodegenerative disorders, particularly Parkinson’s disease [5,40]. HO-1 induction showed a neuroprotective role in in vivo models of Parkinson’s disease [11,41]. Hung et al. utilized an adenovirus containing human HO-1 gene and injected it into rat substantia nigra concomitantly with 1-methyl-4-phenylpyridinium that causes parkinsonism. Overexpression of HO-1 significantly increased the survival rate of dopaminergic neurons, and reduced the production of TNF-α and IL-1β in substantia nigra [11]. Another in vivo study revealed that intracerebral administration of a natural HO-1 inducer also suppressed the dopaminergic neuronal loss and behavioral dysfunction in the 6-OHDA mouse model. Dopaminergic neurons from oxidative stress were protected by upregulation of glial expression of HO-1 [42]. Thus, the increase of HO-1 can be beneficial to suppress neuronal damage and progression of Parkinson’s disease.

### 2.5. Hepatic Fibrosis

Hepatic fibrosis is overly exuberant wound healing that excessive connective tissue builds up in the liver [43]. It is an integral part in chronic liver disease progression, ultimately leading to cirrhosis and hepatocellular carcinoma [44]. HO-1 has been implicated to play an important role in antioxidative stress and cytoprotective systems in the liver. Increased level of HO-1 induced SIRT1 and ameliorated fructose-mediated liver fibrosis by decreasing vascular dysfunction in mice [12]. Cobalt protoporphyrin (CoPP), a HO-1 inducer, was able to suppress oxidative stress markers and negate HO-1 decrease by fructose intake [12]. On the other hand, HO-1 deficiency in mouse models and human caused severe chronic hepatic inflammation, iron deposition and oxidative damage in the liver [45,46]. Most importantly, HO-1 induction reduced liver damage and chronic inflammation by regulating immune cell infiltration or proliferation and TNF receptor signaling in Mdr2 knockout mice, a genetic mouse model of chronic liver inflammation and fibrogenesis [47]. Fibrosis progression was significantly reduced by HO-1 induction with CoPP [47]. Although the therapeutic potential of HO-1 and its mechanism of action are still largely unknown in patients, upregulation of HO-1 gene expression is a potential future clinical implication to improve metabolic balance and attenuate hepatic fibrosis.

## 3. HO-1 Signaling Pathway

**Figure 1:**
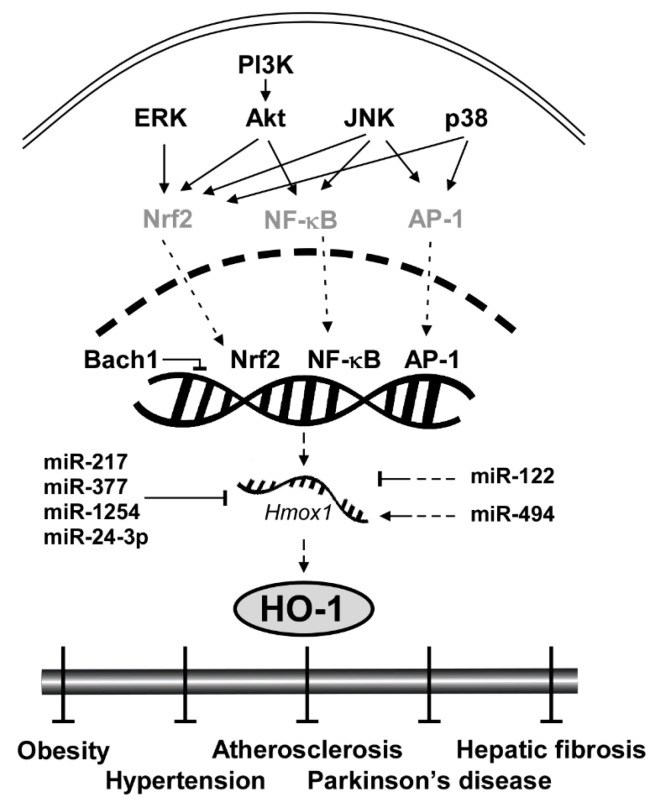
General scheme of signaling pathways in HO-1 induction.

Considering the clinical implications of HO-1 mentioned above, elucidating the signaling pathway of HO-1 and understanding the molecular mechanism are crucial. Here we categorize HO-1 signaling pathway into two segments: upstream kinases and transcription factors of HO-1. Key proteins that regulate transcription factors of HO-1 include extracellular signal-regulated kinase (ERK), c-Jun N-terminal kinase (JNK), p38 and Akt (Figure 1). Transcription factors that bind to upstream of the initiation site of HO-1 to stimulate its mRNA expression include nuclear factor erythroid 2-related factor 2 (Nrf2), nuclear factor kappa-light-chain-enhancer of activated B cells (NF-κB) and activator protein 1 (AP-1). After expressed by the regulator proteins, HO-1 affects downstream elements such as Heme, bilirubin and carbon monoxide (CO).

### 3.1. Upstream Kinases: ERK, JNK, p38 and Akt

ERK, a member of the mitogen-activated protein kinase (MAPK) family, is involved in cellular processes including proliferation [48], differentiation [49] and redox regulation [50]. ERK was reportedly involved in induction of HO-1 by extracellular signals or chemopreventive small molecules [51,52]. Chen and Maines utilized PD98059, a selective ERK pathway inhibitor, and showed that inhibition of ERK blocked HO-1 induction by a nitric oxide donor in a dose-dependent manner [51]. Wu et al. also revealed that pretreatment of PD98059 can prevent HO-1 induction by epigallocatechin-3-gallate (EGCG) [52]. Nrf2 is a well-known transcription factor regulated by ERK to induce HO-1 mRNA expression [53]. Several studies using phytochemicals showed that the activation of the antioxidant regulator, Nrf2, is mediated through ERK phosphorylation [53,54,55].

JNK is another member of MAPK that plays a central role in stress signaling pathways implicated in gene expression, neuronal plasticity and regulation of cellular senescence [56]. Pharmacological inhibitor studies showed that JNK regulates the HO-1 expression level [57]. In rat hepatocytes, the JNK inhibitor SP600125 decreased HO-1 mRNA expression mediated by sodium arsenite [57]. JNK regulates Nrf2 to reduce oxidative stress [58,59]. Pretreatment of SP600125 suppressed Nrf2 translocation under the oxidative stress signal [58]. SP600125 suppressed the JNK signaling pathway and resulted in Nrf2-mediated prevention of diabetic nephropathy [59]. Moreover, JNK also activates NF-κB and Nrf2 [60]. Tsai et al. found that siRNA of JNK inhibited glucose-induced activation of NF-κB in cardiomyocytes [60].

p38, the other member of MAPK family, plays an important role in redox regulation [51], cellular stress response [61] and autophagy [62] via regulating HO-1. An in vitro study using a p38 inhibitor, SB203580, revealed that the inhibition of p38 suppressed the induction of HO-1 by nitric oxide in a dose-dependent manner [51]. Another study also showed that siRNA of p38 MAPK and pretreatment of SB203580 attenuated HO-1 induction in fisetin-stimulated human umbilical vein endothelial cells [63]. The induction of HO-1 by p38 is also correlated with Nrf2 activation. Khayandirobilide A, a natural small molecule with anti-inflammatory property, induces HO-1 expression by p38 MAPK/Nrf2 signaling in RAW264.7 macrophages and BV-2 microglial cells [64].

Akt, a substrate of phosphoinositide 3-kinase (PI3K), is a serine/threonine-specific protein kinase that plays a key role in oxidative damage response [58], cell cycle progression [65] and survival [66]. PI3K-Akt pathway induces HO-1 expression level as a survival signal against oxidative stress-induced injuries [67]. Mo et al. found that hydrogen peroxide enhanced phosphorylation of Akt and that treatment with LY294002, a selective inhibitor of PI3K, suppressed Akt phosphorylation and hydrogen peroxide-induced HO-1 expression [67]. Another study also confirmed the role of the PI3K-Akt pathway on HO-1 expression and further elucidated that Nrf2 is involved in the process. Pretreatment of LY294002 prevented nuclear translocation of Nrf2 and inhibited HO-1 induction in RAW 264.7 cells [58]. In sum, ERK, JNK, p38 and PI3K-Akt pathways govern the HO-1 expression level primarily by regulating important transcription factors such as Nrf2 and NF-κB.

### 3.2. Transcription Factors: Nrf2, NF-κB and AP-1

HO-1 gene (*Hmox1*) is located on chromosome 22q12, and is regulated by several transcription factors including Nrf2, NF-κB and AP-1 [5]. This feature makes HO-1 a converging node in antioxidant mechanism [5] and serves as a critical signaling protein of ferroptosis regulating iron and ROS (reactive oxygen species) homeostasis [68,69,70,71,72]. Nrf2, for instance, is a family member of Cap ‘n’ Collar-basic leucine zipper transcription factor (CNC-bZIP) and is considered as the most pivotal regulator of HO-1 in the brain and nervous system [73]. Without oxidative stress, Nrf2 is located in the cytoplasm by its negative regulator Keap1 that induces ubiquitination and proteasomal degradation of Nrf2 [74]. Under oxidative stress conditions, Keap1 is dissociated from Nrf2 and thus Nrf2 moves into the nucleus, binds to the antioxidant response elements (AREs) sequence of the HO-1 promoter region and initiates the transcription of HO-1 [75]. Once expressed, HO-1 activates a cascade of antioxidant signaling that affects the oxidative status of the cells and protects cells from oxidative challenges [23]. Knockdown experiments confirmed that downregulation of Nrf2 significantly inhibited H-Ras-induced HO-1 transcription [76].

NF-κB and AP-1 also directly regulate HO-1 expression as transcription factors. Unlike evolutionary conserved Nrf2-HO-1 regulation, the HO-1 regulation by NF-κB and AP-1 is dependent on lipopolysaccharide (LPS), a prototypical Toll-like receptor 4 (TLR4) agonist [77]. Once TLR4 is stimulated by LPS or monophosphoryl lipid A, another synthetic TLR4 agonist, it activates NF-κB [78,79] and AP-1 [80]. Inhibition of the NF-κB pathway with small molecules suppressed LPS-induced HO-1 promoter activity [77]. AP-1 homodimers or heterodimers bind to enhancers of the promoter region of *hmox1* [81]. The expression of HO-1 requires AP-1 activation by LPS [80].

### 3.3. Transcription Repressor: Bach1

BTB and CNC homology 1 (Bach1) is ubiquitously expressed in mammalian tissues and functions as a transcriptional suppressor of HO-1 by heterodimerizing with small Maf proteins [82]. While Nrf2 reportedly activates HO-1, Bach1 binds to the enhancers of the *Hmox1* to suppress its expression under normal conditions [83]. Knockout of Bach1 affects oxidative stress damage by HO-1 induction [84]. Bach1 deficiency reduced the severity of osteoarthritis in mice by inducing HO-1 expression [85].

### 3.4. Translational Repressor: miRNAs

MicroRNAs (miRNAs) are a class of small non-coding RNA that govern post-transcriptional gene silencing (PTGS) and affect a wide range of protein expression [86]. There are several miRNAs that downregulate HO-1 both directly and indirectly. miR-217, miR-377, miR-1254 and miR-24-3p directly regulate HO-1 translation. miR-217 and miR-377 in combination showed no change in HMOX1 mRNA levels, but a significant reduction in HO-1 protein expression and enzyme activity [87]. The two miRNAs were able to bind to the 3′ untranslated region (3′ UTR) of human HMOX1 [87]. miR-1254 suppresses HO-1 expression at the post-transcriptional level by directly targeting HO-1 3′ UTR [86]. Pu et al. found that HO-1 expression was inversely correlated with miR-1254 level in human cells [86]. miR-24-3p also targets the 3′ UTR of HO-1 [88]. miR-122 and miR-494 indirectly regulate the HO-1 expression level. Antagomir of miR-122 induced HO-1 mRNA levels in vitro [89]. In contrast, miR-494 upregulates the HO-1 expression level. Endogenous miR-494 inhibition impaired HO-1 induction in response to H_2_O_2_ [90]. The effect of miRNAs on HO-1 is illustrated in Figure 1.

### 3.5. Enzymatic Activity of HO-1

**Figure 2:**
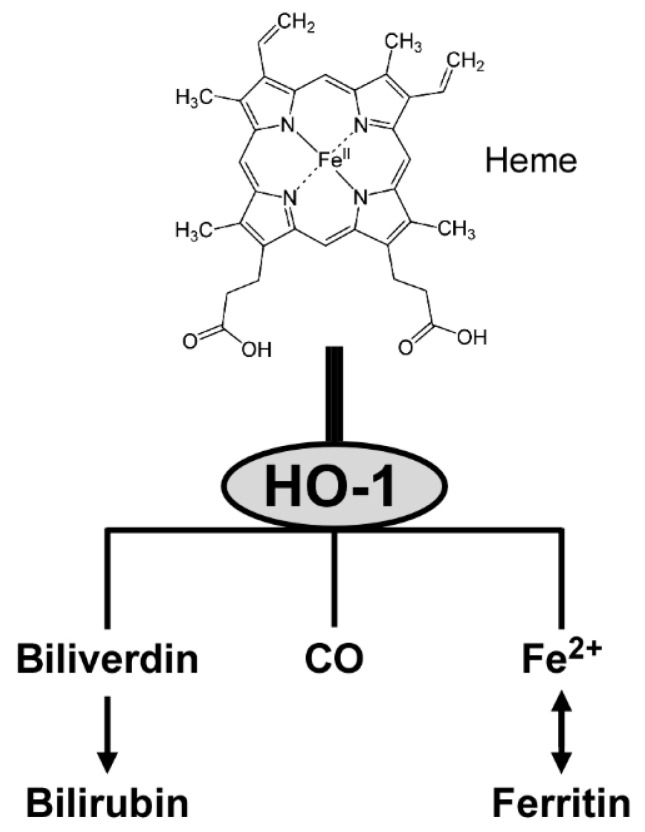
Heme metabolism by HO-1.

Once expressed, HO-1 catalyzes the degradation of heme to yield equimolar amounts of biliverdin, CO and ferrous iron [91]. The three products have anti-inflammatory and antioxidant activities. First, biliverdin is converted to bilirubin that functions as a vasoactive and antioxidant molecule [23]. Second, CO interacts with heme proteins or diffuses to the blood stream, and is transported to the lungs and cleared by exhalation [92]. CO also participates in intracellular signal transduction, including production of anti-inflammatory cytokines and upregulation of antiapoptotic effectors [91]. Third, ferrous iron is an essential requirement for the synthesis of hemoglobin and ferritin, and is involved in cellular redox reactions [23]. Figure 2 provides a conceptual illustration of heme degradation by HO-1.

## 4. Phenolics

Phenolic compounds are present in most food and medicinal plants, and their antioxidant activity and other pharmacological effects have been reported [93]. The antioxidative effect of phenolics in edible sources have been regarded that the phenolics retard oxidative degradation of lipids by direct quenching of ROS [94]. The studies on the molecular mechanism of health promoting effects of phenolics in edible plants that unraveled the bioactivities are also associated with the antioxidative and anti-inflammatory cascades involved with HO-1. The phenolic compounds are the largest family of antioxidative and anti-inflammatory natural products inducing HO-1 to exert a variety of bioactivities including hepatoprotective, cardioprotective, neuroprotective, antiobestic, antidiabetic activity and so on.

### 4.1. Flavonoid

**Figure 3:**
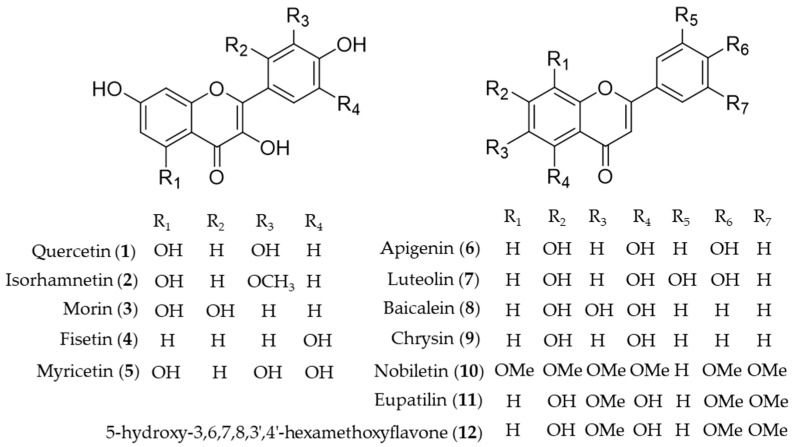
Structures of the flavonoid natural products (**1**–**12**).

Quercetin (**1**) is the most common flavonol present in vegetables, fruits and medicinal herbs with antioxidant and anti-inflammatory activity [95]. Besides direct hydrogen-donating properties to quench ROS, the influence of quercetin on signaling pathways and its indirect interaction with the endogenous antioxidative defense system was investigated. Chen et al. revealed the role of quercetin (**1**) as an inhibitor of iNOS gene expression by inducing HO-1 and mediating the inhibition of IκB kinase, NF-κB and STAT1 [96]. In addition, upregulation of HO-1 expression by activating the nuclear factor erythroid 2 related factor (Nrf2) to bind with ARE in the *ho*-1 gene promoter region was also reported [97,98]. The mechanism of the hepatoprotective effects of quercetin (**1**) on ethanol-induced oxidative damage in hepatocytes was involved in ERK activation and HO-1 upregulation [99]. Later, the signaling pathway of quercetin (**1**) involved in HO-1 induction was revealed as p38 and ERK mediated quercetin (**1**)-derived Nrf2 translocation into nuclei and subsequent induction of HO-1 activity [100]. The hepatoprotective effects of quercetin (**1**) via induction of HO-1 on ethanol-induced microsomal oxidative stress were studied in adult male Sprague-Dawley rats [101]. Quercetin (**1**) also reduces obesity-induced hepatic inflammation by inducing HO-1, which promotes hepatic macrophage polarization in favor of the M2 phenotype [102]. Quercetin (**1**) suppresses microglia-mediated inflammatory responses via the induction of HO-1, and hence protects against obesity-induced hypothalamic inflammation [103]. Under obese conditions, muscle atrophy might be induced by TNFα, but it might be reduced through Nrf2-mediated HO-1 induction accompanied by inactivation of NF-κB of quercetin (**1**) [104]. The therapeutic potential of quercetin (**1**) for inflammatory diseases via enhancement of mitochondrial biogenesis [105], and reduction of NADPH oxidase-derived superoxide generation and oxidative stress [106]. The suppression of hydrogen peroxide (H_2_O_2_)-induced cell damage in endothelial cells by quercetin (**1**) could explain the protective cardiovascular effects of diets rich in the compound [107]. Hayashi et al. reported quercetin (**1**) attenuates oxidative epithelial cell injury in lung inflammation [108]. Another flavonol compound found in common dietary plants, isorhamnetin (**2**) also has HO-1 inducing activity, which results in exhibition of the anti-inflammatory effect [109], attenuation of atherosclerosis [110] and protective effects against oxidative stress-induced cellular damage [111]. A flavanol found in Moraceae plants and many medicinal herbs, morin (**3**) increases induction of HO-1 activity, leading to the anti-inflammatory and antioxidative effects, which implies the potential as a therapeutic for the prevention of neuroinflammation [112] and liver injury [113]. Cytoprotective effect against oxidative stress of fisetin (**4**) treatment resulted from significantly increased Nrf2 nuclear translocation, and ARE-luciferase activity, leading to upregulation of HO-1 expression [63]. Myricetin (**5**) is an anti-inflammatory component that the expression of HO-1 through Nrf2 translocation, found in *Diospyros lotus*, an oriental herbal medicine used for the treatment of diabetes, diarrhea, tumor and hypertension [114]. Apigenin (**6**) and luteolin (**7**) are structurally related flavones easily found in dietary plant sources, which activate Nrf2-ARE-mediated gene expression and induce anti-inflammatory activities with important effects on HO-1 expression [115]. Luteolin (**7**) has been further investigated in mitigation of acute lung injury [116], inhibition of viral-induced inflammatory response [117] and protective effect against renal toxicity [118] via the upregulating Nrf2/ARE/HO-1 pathway. Baicalein (**8**) is the representative bioactive component found in *Scutellaria baicalensis*, an oriental herbal medicine [119]. Its improvement of cardiac contractile function in endotoxemic rats [120] and protective activity for pancreatic β-cells from inflammation [121] might attribute to induction of HO-1 expression. Huang et al. investigated the protective action of three structurally related flavones (chrysin (**9**), apigenin (**6**), and luteolin (**7**)) against oxidative stress in rat primary hepatocytes [122]. Chrysin (**9**), apigenin (**6**) and luteolin (**7**) upregulated the protein expression of HO-1 in a dose-dependent manner and glutamate cysteine ligase catalytic and modifier subunit and increased the intracellular glutathione content and the ratio of glutathione to oxidized glutathione [122]. Nobiletin (**10**), a highly *O*-methylated flavone isolated from citrus peels, significantly induces HO-1 to inhibit NO production and exert anti-inflammatory effects on the crosstalk between adipocytes and macrophages, which implies potential for the prevention of obesity-related metabolic diseases [123]. Eupatilin (**11**), the anti-inflammatory flavone derived from *Artemisia* plants, protects ileal smooth muscle cells (ISMCs) from cell damage caused by indomethacin, and that its cytoprotective action could be attributed to eupatilin-mediated HO-1 induction via ERK and Nrf2 signaling in ISMC [124]. 5-Hydroxy-3,6,7,8,3′4′-hexamethoxyflavone (**12**) from *Hizikia fusiforme* inhibits LPS-stimulated NO production by suppression of iNOS expression and enhancement of HO-1 expression via Nrf2 activation [125]. Structures of the flavonoid natural products (**1**–**12**) are presented in Figure 3.

**Figure 4:**
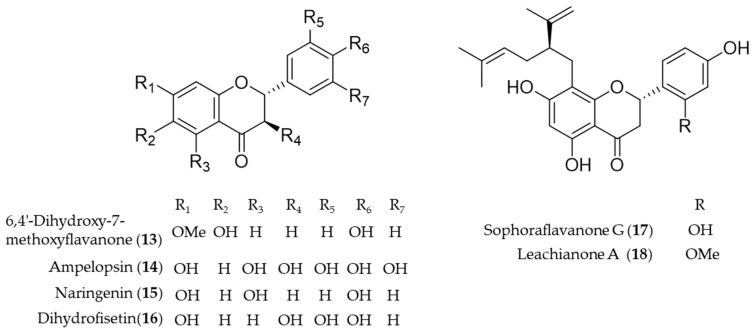
Structures of the flavonoid natural products (**13**–**18**).

Another bioactive *O*-methylated flavone isolated from the heartwood of *Dalbergia odorifera*, 6,4′-dihydroxy-7-methoxyflavanone (**13**) was also proposed as an antioxidative and anti-inflammatory HO-1 inducer in mouse hippocampal HT22 cells and BV2 microglia cells [126]. Ampelopsin (**14**), a flavonoid abundant in Rattan tea (*Ampelopsis grossedentata*), was investigated as a potent antioxidant and neuroprotective agent against H_2_O_2_-induced apoptosis in PC12 cells via upregulation of HO-1 expression [127]. Naringenin (**15**), a flavonone present in various species of citrus fruit, tomatoes and grapes, has anti-inflammatory and antiarthritic properties [128]. Dihydrofisetin (**16**) is a flavanonol, dose-dependently inhibited lipopolysaccharide-induced productions of NO and PGE_2_ in RAW 264.7 macrophage [129]. In addition, dihydrofisetin (**16**) inhibited the activation of MAPK pathway and phosphorylation of IκB-α whereas it upregulated the expression of HO-1 [129]. Sophoraflavanone G (**17**) and leachianone A (**18**) are anti-inflammatory components found in an oriental medicinal herb *Sophora flavescens*, and the compounds belong to a unique and rare class of prenylated flavonoid [130]. The induction of HO-1 by the prenylated flavonoids was identified as the key mechanism of the protective effect against glutamate toxicity in HT22 cells [131]. Sophoraflavanone G (**17**) was also isolated from another allied species, *Sophora alopecuroides*, and the prenylated flavonoid showed potential to treat some inflammatory diseases by targeting PI3K/Akt, JAK/STAT and Nrf2/HO-1 pathways [132]. Structures of the flavonoid natural products (**13**–**18**) are presented in Figure 4.

**Figure 5:**
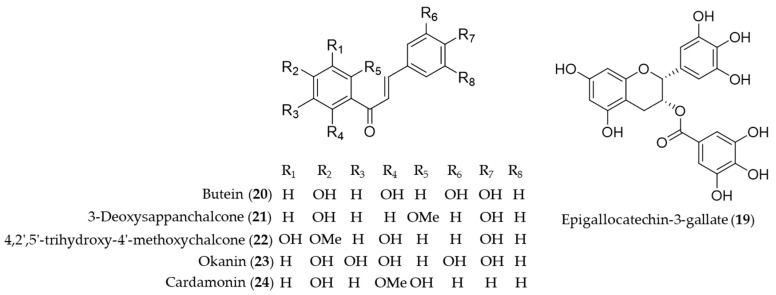
Structures of epigallocatechin-3-gallate (**19**) and the chalcones (**20**–**24**).

Catechins are flavan-3-ol compounds contained in tea as the most abundant phenolic chemical species that exert antioxidant and anti-inflammatory activity [133]. Among the flavonoids classified as catechins in tea, the major green tea catechin, epigallocatechin-3-gallate (**19**, EGCG) has been intensively investigated as it was discovered a HO-1 expression inducing agent that helps protect the neuron against oxidative stress-induced cell death [134], possibly block the pathogenic cycle of Sjögren’s syndrome [135], inhibit inflammatory responses by suppressing the production of proinflammatory cytokines during the adipocyte–macrophage interaction [136], protect against contrast-induced nephropathy by amelioration of oxidative stress and inflammation [137] and mediate beneficial cardiovascular actions via anti-inflammatory actions in vascular endothelium [138]. Chalcones are one of the major classes of natural products with widespread distribution in plant foodstuff with interesting pharmacological activities [139]. Chemically they are characterized of open-chain flavonoids in which the two aromatic rings are joined by a three-carbon α, β-unsaturated carbonyl system [140]. Compared to the relevance in nature, chalcones have been identified as a considerably large group of natural antioxidant and anti-inflammatory agents found in foodstuff or medicinal herbs. Lee et al. reported H_2_O_2_-induced cell death and ROS generation could be inhibited by HO-1 expression induction of butein (**20**) [141]. Based on the role of HO-1 in the development of obesity and insulin resistance, Wang et al. proved that butein (**20**) activates the p38 MAPK/Nrf2/HO-1 pathway to act as a potent inhibitor of adipose hypertrophy and inflammation in a diet-induced obesity mouse model [142]. 3-Deoxysappanchalcone (**21**, also known as isoliquiritigenin 2′-methyl ether) is a major bioactive component isolated from *Caesalpinia sappan*, commonly used herbal medicine for inflammation and to improve blood circulation [143,144]. The molecular mechanism by which 3-deoxysappanchalcone (**21**) exerts anti-inflammatory activity was identified as induction of HO-1 expression at the translational level via activating the AKT/mTOR pathway [145]. 3-Deoxysappanchalcone (**21**) also exhibited antioral cancer effects by HO-1 upregulation via a pathway involving MAP kinases, NF-κB and Nrf2 [146]. The mechanism of anti-inflammatory activity by 4,2′,5′-trihydroxy-4′-methoxychalcone (**22**) from *Dalbergia odorifera* was revealed as inducing the expression of anti-inflammatory HO-1 via the Nrf2 pathway to inhibit proinflammatory mediators such as COX-2 and iNOS [147]. Kil et al. suggested anti-inflammatory action of okanin (**23**) by virtue of its α-β unsaturated carbonyl functional group, reporting that the underlying mechanism is inhibition of NO production and iNOS expression via Nrf2-dependent HO-1 expression [148]. Cardamonin (**24**), a chalcone isolated from *Alpinia katsumadai*, has been investigated to prove anti-inflammatory mechanisms of cardamonin (**24**) is related to the decrease in the level of MDA, iNOS, COX-2, NF-κB and MAPK and induction of the HO-1 expression [149]. Structures of epigallocatechin-3-gallate (**19**) and the chalcones (**20**–**24**) are presented in Figure 5.

**Figure 6:**
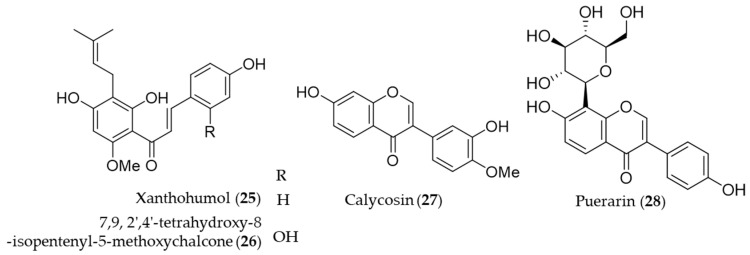
Structures of the prenylated chalcones (**25**–**26**) and the isoflavones (**27**–**28**).

A prenylated chalcone, xanthohumol (**25**) is a major flavonoid contained in hop (*Humulus lupulus*), which is commonly used in beer brewing [150]. Lee et al. reported that xanthohumol (**25**) exerts anti-inflammatory activity through Nrf2-ARE signaling and upregulation of downstream HO-1, to ameliorate inflammatory responses in the brain [151]. Another prenylated chalcone, 7,9,2′,4′-tetrahydroxy-8-isopentenyl-5-methoxychalcone (**26**) isolated from *Sophora flavescens* was identified as an inducer of HO-1 expression, which in turn HO-1 and/or CO suppresses Th2 chemokine expressions induced by cytokines in human HaCaT cells [152]. Calycosin (**27**), an isoflavonoid from the Chinese medicinal herb *Astragalus propinquus,* induces Nrf2 that suppresses the expression of proinflammatory cytokines via p62/Nrf2-linked HO-1 induction in rheumatoid arthritis synovial fibroblasts [153]. The root of *Pueraria lobata* has been used for food and various medicinal purposes in traditional oriental medicine [154]. Puerarin (**28**, daidzein 8-C-glucoside), the main isoflavone glycoside found in *Pueraria lobate*, augments cellular antioxidant defense capacity through estrogen receptor-dependent HO-1 induction via the Gβ1/PI3K/Akt-Nrf2 signaling pathway [155]. Puerarin (**28**) also alleviate the high glucose-induced acute endothelium-dependent vascular dysfunction in rat aortic rings via HO-1 expression induction [154]. Structures of the prenylated chalcones (**25**–**26**) and the isoflavones (**27**–**28**) are presented in Figure 6.

### 4.2. Non-Flavonoid Phenolics

**Figure 7:**
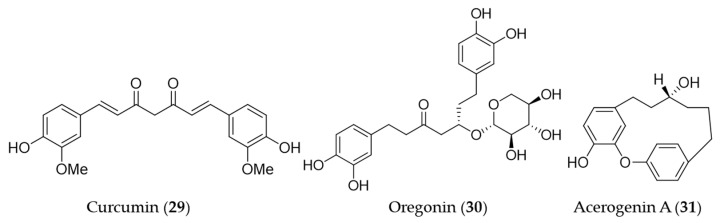
Structures of the diarylheptanoids (**29**–**31**).

Curcumin (**29**) is the main bioactive phenolic compounds present in turmeric, a spice with a variety of medicinal functions including antioxidant, anti-inflammatory [156], antimutagenic and antimicrobial [157,158] activity. A number of research groups has reported the induction of HO-1 expression by curcumin (**29**) and the association with its antioxidant, anti-inflammatory activity, furthermore, the therapeutic and preventive potential for the related diseases since the first report on its HO-1 inducing activity [159]. The early investigations on induction of HO-1 by curcumin have markedly contributed to extending the understanding of the molecular mechanism of antioxidant effects exerted by HO-1. Curcumin (**29**) was identified as a potent inducer of HO-1 in vascular endothelial cells and the increased heme oxygenase activity is an important component in curcumin-mediated cytoprotection against oxidative stress [160]. Hill-Kapturczak et al. elucidated the mechanism of HO-1 induction by curcumin (**29**) was involved in the NF-κB pathway in human renal cells [161]. In the course of the study on antioxidant potential of curcumin (**29**), it was discovered that stimulation of *ho*-1 gene activity could promote inactivation of the Nrf2–Keap1 complex, leading to increased Nrf2 binding to the resident *ho*-1 AREs [162]. McNally et al. discovered that PKC and p38 MAPK activity are required for full induction of HO-1 in the course of their study using curcumin (**29**) [163]. On the basis of the ethnopharmacological background [164,165,166], hepatoprotective effects by curcumin (**29**) pretreatment was involved in the dose- and time-dependent induction of HO-1 [167]. Furthermore, the HO-1 induction exhibited inhibition of hepatitis C virus (HCV) replication along with AKT pathway inhibition [168]. The induction of HO-1 expression by curcumin (**29**) may protect human retinal pigment epithelial cells against oxidative stress by reducing ROS levels [169]. The recent study on the hepatoprotective potential of curcumin demonstrated that hepatic chronic inflammation could be ameliorated through the activation of HO-1 by curcumin (**29**) [170]. Cisplatin is a standard chemotherapeutic agent for solid malignances, in spite of the high incidence of side effects including ototoxicity [171]. Fetoni et al. reported that curcumin (**29**) treatment attenuated hearing loss induced by cisplatin via curcumin-mediated upregulation of HO-1 [172]. Curcumin (**29**) also protected SH-SY5Y cells against appoptosin-induced intrinsic caspase-dependent apoptosis by upregulating HO-1, attenuating accumulation of intracellular heme and ROS [173]. Oregonin (**30**) is a glucose-conjugated diarylheptanoid, which shares the same structural backbone with curcumin (**29**). Oregonin (**30**) is known as the antioxidant and anti-inflammatory agent isolated from leaves of *Alnus formosana* [174], and the bioactivities are also involved in the induction of HO-1 [175]. A diarylheptanoid with cyclic structure, acerogenin A (**31**) from Japanese folk medicine *Acer nikoense*, showed neuroprotective effects and ROS reduction on glutamate-induced neurotoxicity by inducing the expression of HO-1 [176]. Structures of the diarylheptanoids (**29**–**31**) are presented in Figure 7.

**Figure 8:**
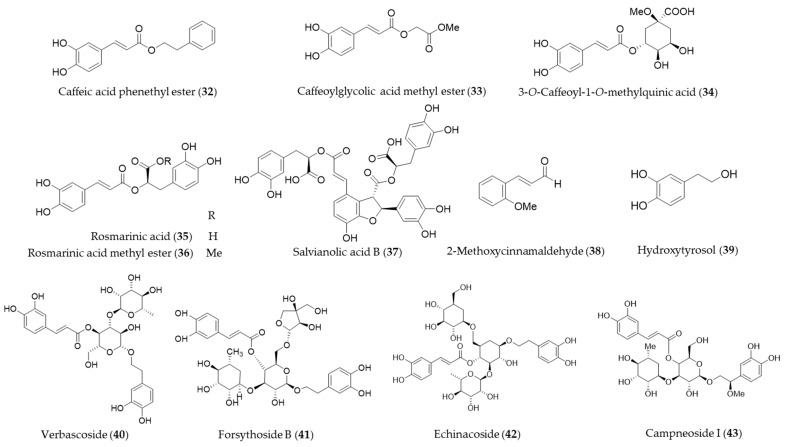
Structures of the phenolic natural products (**32**–**43**).

Caffeic acid phenethyl ester (**32**, CAPE) is a hydrophobic natural phenolic compounds with a variety of bioactivities found in a wide range of plants and honeybee propolis [177,178]. CAPE (**32**) is one of the first few natural products identified as an inducer of HO-1 expression [179], and a number of follow-up studies on CAPE (**32**) involving HO-1 induction and the health promoting-potential have been published. Wang et al. proposed the cytoprotective potential of CAPE (**32**) against menadione-induced oxidative stress in human umbilical vein endothelial cells (HUVEC) via upregulation of HO-1 by CAPE (**32**) [180]. Interestingly, caffeic acid, a potential metabolite of CAPE (**32**) with similar free radical scavenging ability, however, did not show any cytoprotective effect nor induce HO-1 in the study [180]. Morroni et al. suggested that CAPE (**32**) could potentially be considered as a promising neuroprotective agent against progressive neurodegenerative diseases, demonstrating administration of CAPE (**32**) counteracted oxidative stress accompanied by an induction of Nrf2 and HO-1 in a mouse model [181]. Kurauchi et al. also investigated the neuroprotective potential of CAPE (**32**) in vivo, examining the expression of HO-1 and the brain-derived neurotrophic factor [182]. High-level glucose-mediated oxidative stress could be attenuated by HO-1 induction by CAPE (**32**), and it may be useful in diabetes and other stress-induced pathological conditions [183]. CAPE (**32**) is putatively biosynthesized from caffeic acid and phenethyl alcohol, and it is not difficult to perform studies on chemical synthesis of derivatives and structure–activity relationship (SAR). Thus, SAR studies for enhancement of HO-1 induction using synthetic derivatives of CAPE (**32**) were reported as well [183,184,185]. A caffeic acid derivatives, caffeoylglycolic acid methyl ester (**33**) [89] contained in grains of *Sorghum bicolor* and 3-*O*-caffeoyl-1-methylquinic acid (**34**) [186] from bamboo leaves exerted anti-inflammatory effects by inducing HO-1 expression. Another type of caffeic acid ester derivative found in rosemary and other foodstuff, rosmarinic acid (**35**) [187] and a derivative isolated from *Perilla frutescens,* rosmarinic acid methyl ester (**36**) [188] also exert their antioxidant and anti-inflammatory action via induction of HO-1 expression. Salvianolic acid B (**37**) is a polyphenolic compounds isolated from *Salvia miltiorrhiza* (Danshen), a traditional oriental medicinal herb, which improves vascular function by inhibiting inflammatory responses and promoting endothelium-dependent vasodilation via induction of HO-1 expression [189]. 2-Methoxycinnamaldehyde (**38**) is another phenylpropanoid natural product found in *Cinnamomum cassia,* which has been used as a spice and medicinal herb for inflammatory disorders. 2-Methoxycinnamaldehyde (**38**) possibly protects from myocardial I/R-injury due to antioxidant and anti-inflammatory action by HO-1 induction [190]. Hydroxytyrosol (**39**) is commonly found in olive oil and leaves with even stronger antioxidant potential than other natural phenolics but gallic acid [191]. The cytoprotective action against oxidative injury promotion and wound healing in vascular endothelial cells by hydroxytyrosol (**39**) could be explained with Nrf2 activation and HO-1 induction [192,193]. Glycosides of phenylpropanoids, verbascoside (**40**), forsythoside B (**41**), echinacoside (**42**) and campneoside I (**43**) also reported as HO-1 inducing agents [194]. Structures of the phenolic natural products (**32**–**43**) are presented in Figure 8.

**Figure 9:**
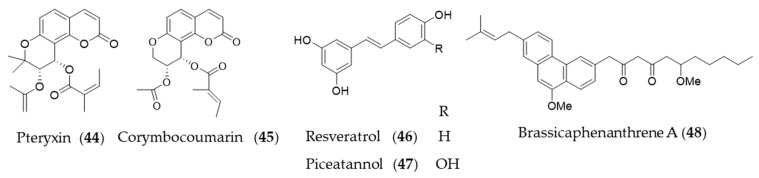
Structures of the phenolic natural products (**44**–**48**).

Coumarins are a group of phenolic natural products composed of fused benzene and α-pyrone rings biosynthesized from cinnamic acid via ortho-hydroxylation, trans-cis isomerization of the side-chain double bond and lactonization [195]. *Peucedanum japonicum* has been used as a folk medicine in East Asia, and the antioxidant and antityrosinase active compounds were found in the leaf extract [196,197]. A coumarin derivative, pteryxin (**44**) isolated in *P. japonicum* was identified as HO-1 inducing agent through Nrf2-ARE signaling [198]. Another coumarin derivative, corymbocoumarin (**45**) from *Seseli gummiferum* subsp. *corymbosum* was investigated for its anti-inflammatory effect through suppression of NF-κB signaling pathway and induction of HO-1 expression [95]. Resveratrol (**46**) is a polyphenolic stilbene that is frequently found in grapes and other food products, especially well-known as principal active component of red wine and grape peel [199,200]. The ability of resveratrol (**46**) to attenuate proinflammatory cytokine expression was investigated [201], and then potential of resveratrol (**46**) to induce HO-1 expression to exert antioxidant and anti-inflammatory action was suggested [202]. Resveratrol (**46**) increased the level of nuclear Nrf2/ARE reporter activity to induce HO-1 expression, which exerts a preventive effect on vascular occlusive diseases [175,203], potential neuroprotective action [204,205] and protective effect on cardiomyocyte apoptosis [206]. Piceatannol (**47**), structurally almost identical to resveratrol (**46**), with the exception of an additional hydroxyl group at the 3′-carbon is also a phytochemical inducer of HO-1 expression [207]. Brassicaphenanthrene A (**48**) isolated from common *Brassica rapa* (turnip) apparently does not look related with resveratrol, but brassicaphenanthrene A (**48**) and resveratrol (**46**) both belong to stilbenoid. Brassicaphenanthrene A (**48**) was also identified as a phytochemical inducer of HO-1 expression [208]. Structures of the phenolic natural products (**44**–**48**) are presented in Figure 9.

**Figure 10:**
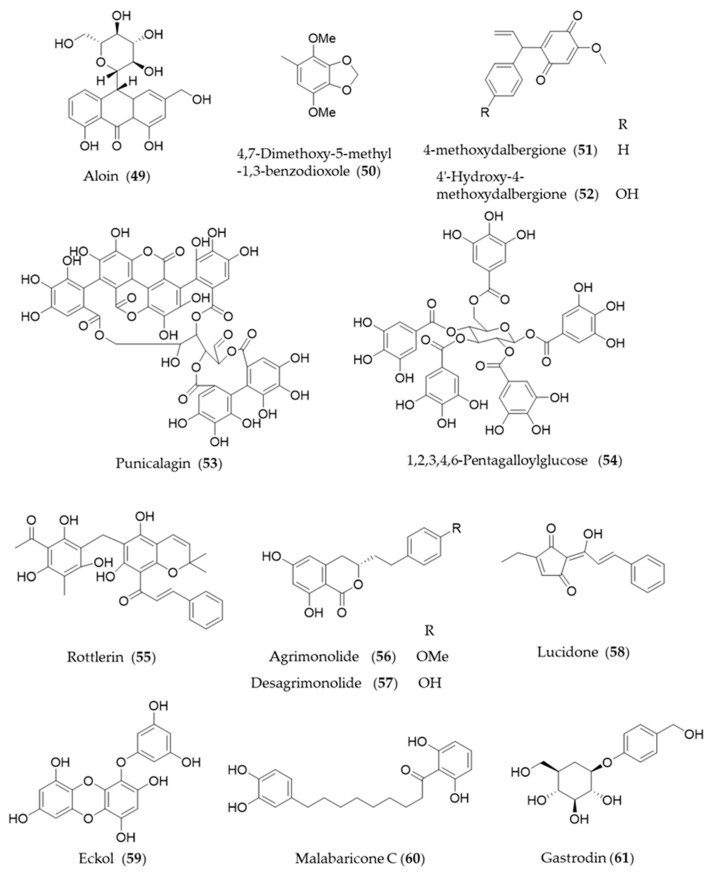
Structures of the phenolic natural products (**55**–**61**).

Aloin (**49**) is the major anthraquinone glycoside obtained from the *Aloe* species and exhibits anti-inflammatory and antioxidative activities via HO-1 induction and reduced NF-κB-luciferase activity [209]. A hydrophobic benzenoid isolated from *Antrodia camphorate* a mushroom used for pharmaceutical purpose, 4,7-dimethoxy-5-methyl-1,3-benzodioxole (**50**) has potential anti-inflammatory activity via increased HO-1 expression that attenuated the LPS-induced proinflammatory factors and iNOS and TLR4 protein levels [210]. Another type of hydrophobic phenolic derivatives, 4-methoxydalbergione (**51**) and 4′-hydroxy-4-methoxydalbergione (**52**) from *D. odorifera* exhibited HO-1 induction to exert anti-inflammatory and cytoprotective effects [211]. Punicalagin (**53**), an ellagitannin polyphenol found in *Punica granatum* (pomegranates) with antioxidant activity had protective effects on H9c2 cardiomyocytes from doxorubicin-induced toxicity [212] and human retinal pigment epithelium cells from UV radiation-induced oxidative damage [213] through activation of Nrf2/HO-1 signaling. Another gallic acid derivative from *P. granatum*, 1,2,3,4,6-pentagalloylglucose (**54**) is the pentagallic acid ester of glucose that also induces the expression of HO-1 in the PC12 cells and its regulation in the PC12 cells [214]. Rottlerin (**55**), isolated from *Mallotus philippinensis*, was originally reported to inhibit PKC δ [215], but it seems to induce upregulation of HO-1 via PKC δ-independent pathway [216]. The bioactive polyphenols in agrimony, agrimonolide (**56**) and desmethylagrimonolide (**57**) induce HO-1 expression, which can be regulated partially by the blockade of p38 MAPK signaling pathway and inhibiting nuclear translocation of Nrf2 [217]. Lucidone (**58**) from the fruits of *Lindera erythrocarpa*, significantly induced HO-1 production and led to the increase of its production of biliverdin for inducing an antiviral interferon response and inhibiting HCV NS3/4A protease activity [218]. A phlorotannin found in an edible alga *Ecklonia cava*, eckol (**59**) attenuates oxidative stress by activating Nrf2-mediated HO-1 induction via extracellular regulated kinase (Erk) and PI3K/Akt signaling [219]. Malabaricone C (**60**) is known to exert a variety of pharmacological activities of nutmeg, and inhibits platelet-derived growth factor-induced proliferation and migration of aortic smooth muscle cells through induction of HO-1 [220]. The phenolic glucoside gastrodin (**61**), the bioactive component of Chinese herbal medicine *Gastrodia elata*, has been known to display antioxidant activity induce HO-1 expression to exert a cytoprotective role in the dopaminergic cell culture system [221], and alleviate H_2_O_2_-induced oxidative stress in mouse liver [222]. Structures of the phenolic natural products (**55**–**61**) are presented in Figure 10.

## 5. Terpenoids and Steroids

### 5.1. Monoterpenes

**Figure 11:**
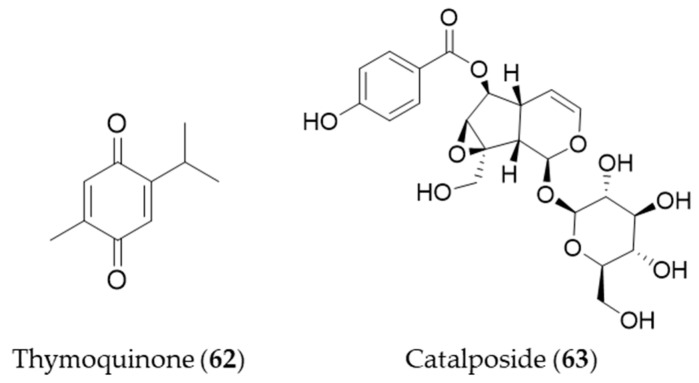
Structures of the monoterpenes (**62**–**63**).

Thymoquinone (**62**) is an active constituent that belongs to monoterpenoid isolated from *Nigella sativa* that possesses alkylated benzoquinone structure [223]. Thymoquinone (**62**) induces HO-1 expression in HaCaT cells by activating Nrf2 through ROS-mediated phosphorylation of Akt and AMPKα [224]. Catalposide (**63**) belongs to a group of modified monoterpenes, iridoid glycoside that possesses antimicrobial, antitumoral and anti-inflammatory properties. Catalposide (**63**) isolated from the stem bark of *Catalpa ovata* is a potent inducer of HO-1 that mediates cytoprotection against oxidative damage [225]. Structures of the monoterpenes (**62**–**63**) are presented in Figure 11.

### 5.2. Sesquiiterpenes

**Figure 12:**
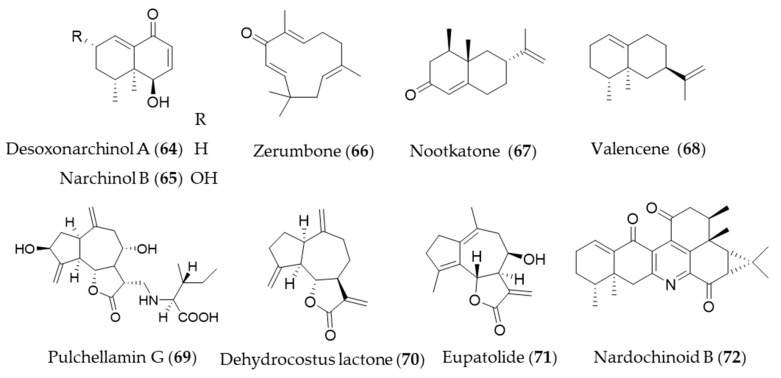
Structures of the sesquiterpenes (**64**–**72**).

Desoxonarchinol A (**64**), isolated form *Nardostachys jatamansi*, is an effective inducer of HO-1, which regulates neutrophil infiltration in acute pancreatitis via chemokine (C-X-C motif) ligand 2 inhibition [226]. Kim et al. investigated the antineuroinflammatory effects via upregulation of Nrf2/HO-1 signaling by desoxonarchinol A (**64**) along with another derivative narchinol B (**65**) [227]. Zerumbone (**66**) is a monocyclic sesquiterpene and the major active phytochemical compound in *Zingiber zerumbet* [228] Zerumbone (**66**) is known to have antioxidant activity, anti-inflammation, immunomodulatory effect and anticancer activity [11], and Leung et al. suggested the protective mechanisms of zerumbone (**66**) on acute lung injury were exerted via upregulation of Nrf2/HO-1 signaling [229]. Shin et al. reported topical application of zerumbone (**66**) onto dorsal skin of hairless mice induces activation of Nrf2/HO-1 expression that provides chemopreventive effects on mouse skin carcinogenesis [230]. *Cyperus rotundus* has been used as traditional folk medicine for the treatment of inflammatory diseases, and the possible anti-inflammatory mechanism is, at least, due to HO-1 induction, in which sesquiterpenes such as nootkatone (**67**) and valencene (**68**) play a crucial role [231]. Pulchellamin G (**69**) is an amino acid-sesquiterpene lactone conjugate isolated from *Saussurea pulchella*, and the anti-inflammatory activity was associated with induction of HO-1 expression [232]. Jeong et al. suggested the α-methylene-γ-butyrolactone moiety in dehydrocostus lactone (**70**) is crucial for cytoprotective HO-1 expression through activation of the Nrf2 [233], and Park et al. suggested that dehydrocostus lactone (**70**) might be useful for the treatment of sepsis through the mechanism [234]. Eupatolide (**71**), a sesquiterpene lactone from *Inula britannica* could suppress platelet-derived growth factor-induced proliferation and migration of vascular smooth muscle cells (VSMCs) through HO-1 induction via the ROS-Nrf2 pathway and may be a potential HO-1 inducer for preventing or treating vascular diseases [235]. Nardochinoid B (**72**) is a terpene composed of thirty carbons and a nitrogen, but not a triterpene. The putative biosynthetic pathway of nardochinoid B (**72**) is dimerization of nardosinane sesquiterpenes, and it exerts significant anti-inflammatory activity [236]. The mechanism of anti-inflammatory action by nardochinoid B (**72**) was revealed as activating the Nrf2/HO-1 pathway [237]. Structures of the sesquiterpenes (**64**–**72**) are presented in Figure 12.

### 5.3. Diterpenes

**Figure 13:**
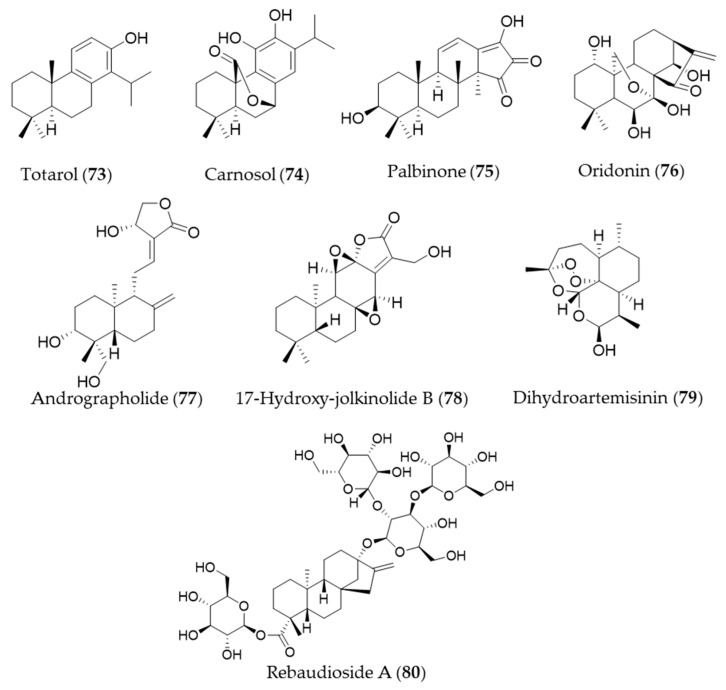
Structures of the diterpenes (**73**–**80**).

A diterpenoid isolated from the sap of *Podocarpus totara*, totarol (**73**) is known for its potent antimicrobial activity [238]. The neuroprotective activity of totarol (**73**) by increased Akt and GSK-3β phosphorylation, Nrf2, HO-1 expressions was investigated in a model of acute cerebral ischemic injury in the rat [239]. Another phenolic diterpene, carnosol (**74**), could target *ho*-1 to induce HO-1 expression, and increased the nuclear levels of Nrf2 [240,241]. The bioactive diterpene, palbinone (**75**) in *Paeonia suffruticosa,* which has been traditionally employed for vitalizing blood circulation and alleviating liver and inflammatory diseases could induce HO-1 expression in the hepatic cells [242]. Oridonin (**76**) is an *ent*-kaurene diterpene with an immunosuppressive effect isolated from *Isodon serra* [243]. According to the study performed by Hu et al., oridonin (**76**) had a distinct effect on promoting CD4+/CD25+ Treg differentiation and modulating Th1/Th2 balance, and this effect may be achieved via inducing the anti-inflammatory target HO-1 [244]. Andrographolide (**77**) is a well-known diterpene, characterized of labdane diterpenoid with a five-membered unsaturated lactone moiety, isolated from an oriental medicinal herb *Andrographis paniculata* [245]. Yu et al. suggested that stimulation of Nrf2-dependent HO-1 expression is involved in the suppression of TNF-α-induced ICAM-1 expression exerted by andrographolide (**77**) [246]. Another diterpenoid with a five-membered lactone isolated from *Euphorbia fischeriana,* 17-hydroxy-jolkinolide B (**78**) could inhibit COX-2, iNOS in a concentration-dependent manner. These inhibitory effects were caused by suppression of MAPK phosphorylation and NF-κB activation and HO-1 induction [247]. Dihydroartemisinin (**79**) is isolated from *Artemisia annua* that has prominent immunomodulatory activity that regulates the Th/Treg balance by inducing activated CD4+ T cell apoptosis via HO-1 induction in mouse models of inflammatory bowel disease [248]. Rebaudioside A (**80**) is a commercially used natural sweetener from *Stevia rebaudiana*, which has been discovered as a potential candidate hepatoprotective agent that activate Nrf2/ARE, and the expression of HO-1 and NAD(P)H quinone oxidoreductase 1 (NQO1) [249]. Structures of the diterpenes (**73**–**80**) are presented in Figure 13.

### 5.4. Titerpenes

**Figure 14:**
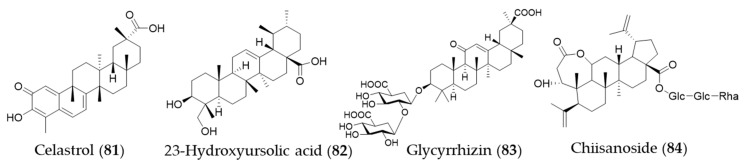
Structures of the triterpenes (**81**–**84**).

Celastrol (**81**) is a triterpene isolated from the plant family Celastraceae and these plants have been used in traditional Chinese medicine for their anti-inflammatory property [250]. Yu et al. investigated the ability of celastrol (**81**) to attenuate hypertension-induced inflammation and oxidative stress in VSMCs via HO-1 induction [251]. The celastrol-mediated HO-1 expression may reduce HIV-1 Tat-induced neuroinflammatory responses [252], HCV replication [253] and macrophage M1 polarization [254]. Jeong et al. discovered an anti-inflammatory phytochemical, an ursane-type triterpene, 23-hydroxyursolic acid (**82**, 3β, 23-dihydroxyurs-12-en-28-oic acid) from flowered fruit-spike of *Prunella vulgaris*, which increased the expression of HO-1 in a dose-dependent manner in human liver-derived HepG2 cells [255]. Another ursane-type triterpene from *Cucurbita pepo* was also found as an inducer of HO-1 expression [256]. The fruiting bodies of *Ganoderma lucidum* (commonly known as the Reishi mushroom) are widely used in China, Japan and Korea as a valuable crude drug, especially in the treatment of chronic hepatitis, nephritis, hepatopathy, neurasthenia, arthritis, bronchitis, asthma, gastric ulcer and insomnia [257]. Lanostane-type (tetracyclic) triterpenes were identified as phytochemicals responsible for the anti-inflammatory activity inducing HO-1 expression [258]. Glycyrrhizin (**83**) is a triterpene glycoside, which is responsible for sweet taste and pharmacological activity of *Glycyrrhiza glabra* (licorice) [259]. Kim et al. proposed that glycyrrhizin (**83**) reduces high mobility group box 1 (HMGB1) secretion by p38/Nrf2-dependent induction of HO-1, which may prevent sepsis [259]. Mou et al. demonstrate that glycyrrhizin (**83**) protects human melanocytes from H_2_O_2_-induced oxidative damage via the Nrf2-dependent induction of HO-1, providing evidence for the application of glycyrrhizin (**83**) in the treatment of vitiligo [260]. Another triterpene glycoside chiisanoside (**84**) isolated from *Acanthopanax sessiliflorus* showed a hepatoprotective effect via an antioxidative effect and inflammatory suppression in NF-κB and activation of Nrf2/HO-1 signaling [261]. Structures of the triterpenes (**81**–**84**) are presented in Figure 14.

### 5.5. Steroids

**Figure 15:**
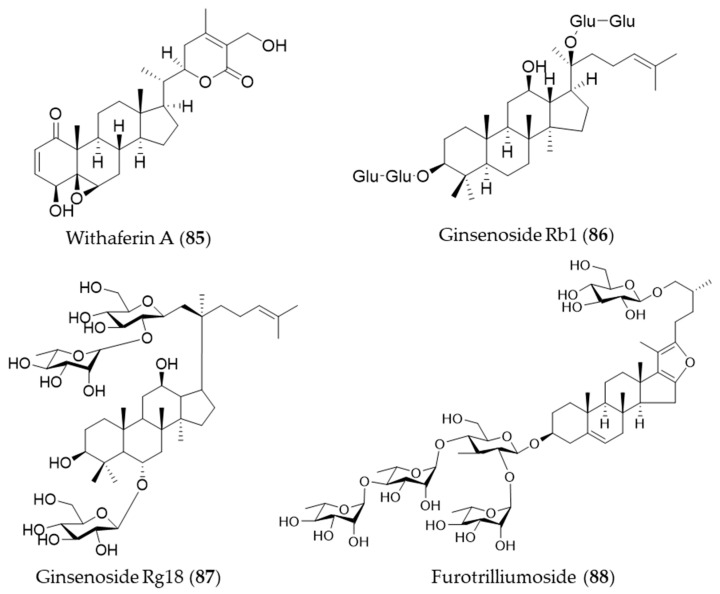
Structures of the steroids (**85**–**88**).

Withaferin A (**85**) is one of bioactive steroidal phytochemicals, withnolides, which is responsible for the bioactivities of *Withania somnifera*, also known as “Ashwagandha”, “Indian ginseng” or “winter cherry”, a frequently used medicinal herb in Ayurvedic medicine (Indian traditional medicine) [262]. Withaferin A (**85**) induces HO-1 expression in endothelial cells via upregulation and increased nuclear translocation of Nrf2 in a time- and concentration-dependent manner [262]. Ginsenosides are unique steroid glycosides and triterpene saponins that are present exclusively in the genus *Panax* [263]. More than 150 natural ginsenosides have been reported [264], and they share a common tetracyclic structure, but the number and position of sugar or hydroxyl moiety may vary among ginsenosides [265]. Ginsenoside Rb1 (**86**) was reported to have a protective effect against oxidative stress by increasing HO-1 expression through an estrogen receptor-related PI3K/Akt/Nrf2-dependent pathway in human dopaminergic cells [266]. Ginsenoside Rg18 (**87**) attenuated neuroinflammation in BV2 microglia and amyloid-β-induced oxidative stress in SH-SY5Y neurons via Nrf2/HO-1 induction [267]. Another type of steroidal glycoside, furotrilliumoside (**88**), isolated from *Trillium tschonoskii* also upregulated HO-1 expression via Nrf2 that might act as a natural agent to treat inflammatory diseases [268]. Structures of the the steroids (**85**–**88**) are presented in Figure 15.

### 5.6. Other Natural Products from Mevalonate Pathway

**Figure 16:**
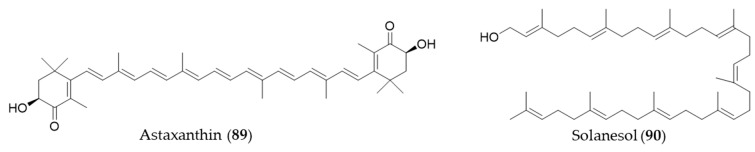
Structures of astaxanthin (**89**) and solanesol (**90**).

Astaxanthin (**89**) is a blood-red pigment that belongs to xanthophyll, which is present in many aquatic organisms such as krill, algae, shrimp, salmon and so on [269]. It is produced commercially from large cultures of microalga, *Haematococcus pluvialis* as an ingredient for dietary supplements and fish feeds [176]. Astaxanthin (**89**) could also ameliorate the chemotherapeutic drug, doxorubicin-induced liver injury through the Keap1/Nrf2/HO-1 pathway in mice [270]. Solanesol (**90**) is classified as a nonaisoprenoid, and it is known to be present in tobacco, potato and tomato [271]. Yao et al. suggested that the anti-inflammatory activity of solanesol (**90**) also comes from induction of expression of HO-1 via p38 and Akt and suppression of proinflammatory cytokines production [272]. Structures of astaxanthin (**89**) and solanesol (**90**) are presented in Figure 16.

## 6. Lipids

**Figure 17:**
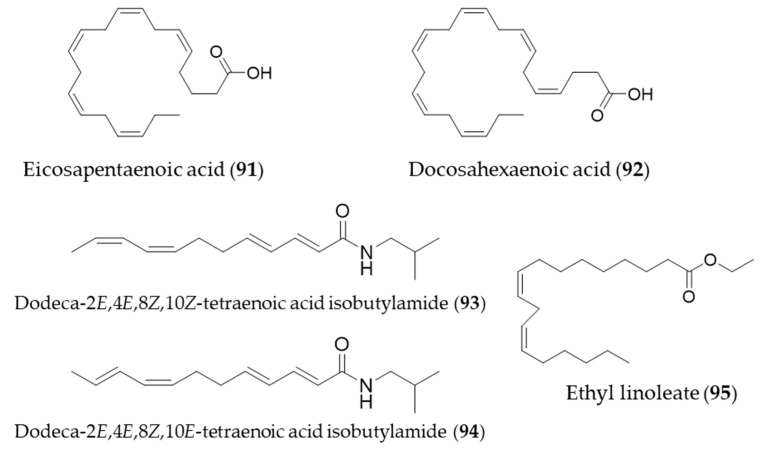
Structures of the lipid natural products (**91**–**95**).

The bioactive natural products with HO-1 induction that belong to lipids were small in number. Interestingly, they were all unsaturated fatty acids or fatty acid derivatives. One of the omega-3 essential fatty acids, eicosapentaenoic acid (**91**, EPA) protects against H_2_O_2_-induced oxidative stress in endothelial cells by activating Nrf2 and inducing HO-1 expression [273]. Another omega-3 fatty acid, docosahexaenoic acid (**92**, DHA) also increased HO-1 expression in U937 cells via activation of ERK1/2 and increased Nrf-2 binding to ARE [274]. The major alkamides dodeca-2*E*,4*E*,8*Z*,10*Z*(*E*)-tetraenoic acid isobutylamides (**93**, **94**), isolated from *Echinacea purpurea* have potential for prevention of acute hepatic injury through JNK pathway-mediated HO-1 expression [275]. Ethyl linoleate (**95**) from garlic was also found to attenuate proinflammatory cytokine production by inducing HO-1 [276]. Structures of the lipid natural products (**91**–**95**) are presented in Figure 17.

## 7. Lignan

**Figure 18:**
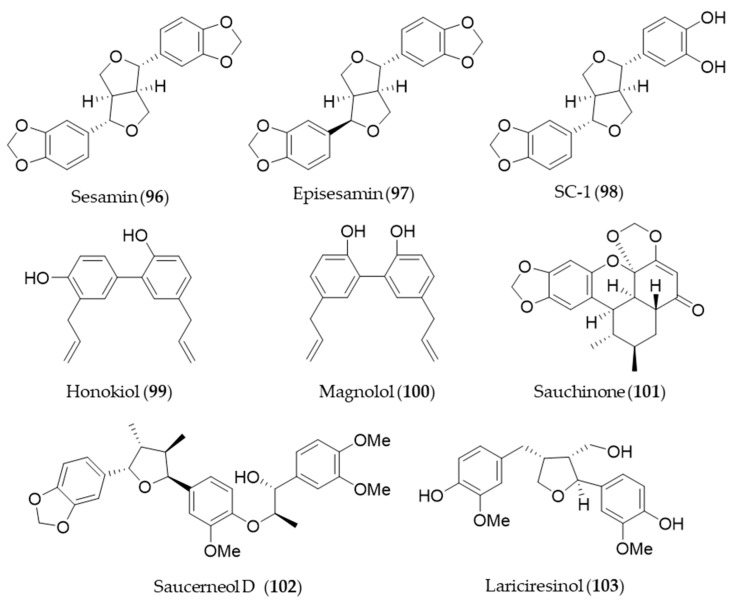
Structures of the lignan natural products (**96**–**103**).

Sesamin (**96**) and its stereoisomer episesamin (**97**) are bioactive lignans found in sesame oil [277]. They are readily metabolized by cytochrome P-450 to yield a series of metabolites, and the metabolites were screened for antioxidant potential [278]. The most potent antioxidant metabolite, SC-1 (**98**) was capable of protecting against oxidative stress-induced neuronal cell death in part through induction of HO-1 via Nrf2/ARE activation [278]. Honokiol (**99**), a phenolic lignan originally isolated from *Magnolia obovate*, significantly inhibited cyclosporine A-induced and Ras-mediated survival of renal cancer cells through the downregulations of the vascular endothelial growth factor (VEGF) and HO-1 [279]. It implies honokiol (**99**) may help to prevent tumor-promoting effects of an immunosuppressant drug, cyclosporine A in transplant patients [279]. Another phenolic lignan isolated from *Magnolia officinalis,* magnolol (**100**) inhibits *Porphyromonas gingivalis* LPS-induced inflammation in macrophages, which is mediated by HO-1 activation, and thereby it is plausible for treatment of periodontitis [280]. *Saururus chinensis*, an oriental medicinal herb has been used to treat jaundice, pneumonia, edema, fever and several inflammatory diseases [281]. Sauchinone (**101**), a diastereomeric lignan isolated from *Saururus chinensis* protects vascular inflammation [282] and significantly inhibit NO production and inflammatory mediators expression [283] via HO-1 induction. Another lignan isolated from *Saururus chinensis*, saucerneol D (**102**) suppresses LPS-induced activation of dendritic cells through the induction of HO-1 [284]. Lariciresinol (**103**), isolated from *Rubia philippinensis*, has a dimeric structure of a phenylpropanoid with a core structure of tetrahydrofurano ring, and exerts potent antioxidant activity [285]. The antioxidant potential of lariciresinol (**103**) is due to the increased transcriptional and translational levels of antioxidant enzymes by activating Nrf2-mediated HO-1 induction via p38 signaling [286]. Structures of the lignan natural products (**96**–**103**) are presented in Figure 18.

## 8. Amino Acid Derivatives

**Figure 19:**
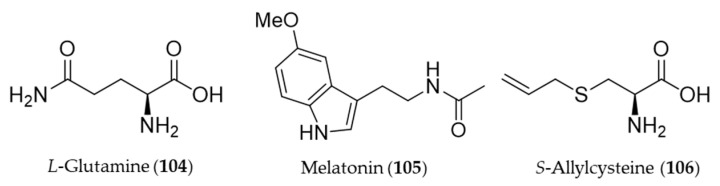
Structures of the amino acid derivatives (**104**–**106**).

*L*-Glutamine (**104**) reduced colonic damage in colitis by the mechanism of the protection associated with HO-1 induction effects, which were documented by the decrease in NF-κB expression, MDA, and caspase-3 levels and concurrent increase in GSH levels and HO-1 overexpression in the colonic tissue [287]. Melatonin (**105**) is a neurohormone derived from an amino acid tryptophan and released by the pineal gland to regulate the circadian rhythm [288]. In vertebrates, melatonin (**105**) is produced in darkness, by the pineal gland [289], but it is also ingested from plant foodstuff such as bananas, grapes, rice, herbs, plums and olive [290]. Beside the circadian regulation, melatonin (**105**) can prevent damages of cells from oxidative stress, especially involved with neurodegeneration in aging and Alzheimer’s disease [291,292]. Clapp-Lilly et al. suggested that melatonin (**105**) induced redox active iron and HO-1 immunoreactivity that it may be a potential therapeutic agent in the prevention of oxidative stress associated with Aβ and Alzheimer’s disease [293]. The HO-1 induction by melatonin (**105**) also potentiates the neuroprotective effect of resveratrol against oxidative injury [294], and inhibits type 1 interferon signaling of TLR4 in hepatic ischemia/reperfusion [295]. Garlic yields a variety of organosulfuric compounds with health benefits [296]. An unique amino acid derivative present in raw garlic, *S*-allylcysteine (**106**) provided potent anti-inflammatory, antioxidative and mucosa protective effects against nonsteroidal anti-inflammatory drug (NSAID)-induced damages via induction of HO-1 [296]. Structures of the amino acid derivatives (**104**–**106**) are presented in Figure 19.

## 9. Alkaloids and Nitrogen-Containing Natural Products

**Figure 20:**
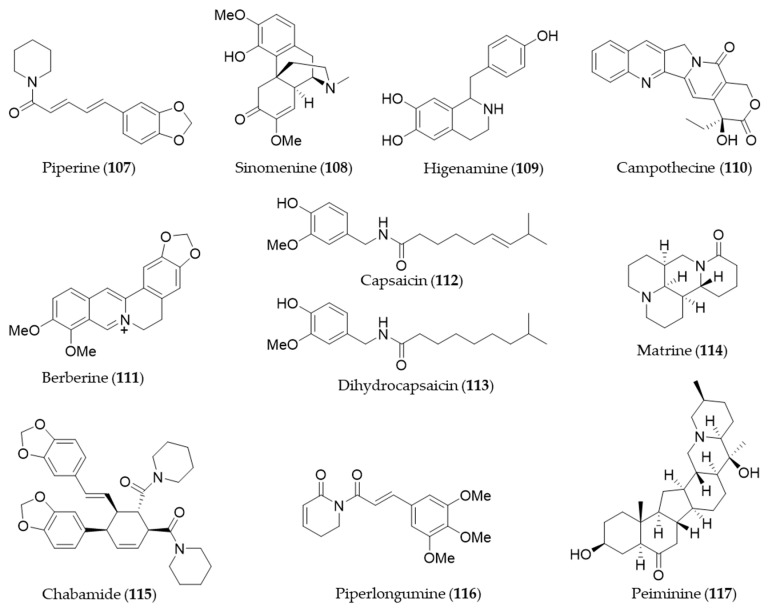
Structures of the alkaloids and nitrogen-containing natural products (**107**–**117**).

Piperine (**107**) is a major alkaloid present in black pepper (*Piper nigrum*), which is known to possess pharmacological benefits, such as antimicrobial, antipyretic and anti-inflammatory effects [173]. The expression of HO-1 by piperine (**107**) is mediated by both JNK pathway and Nrf2, and the expression inhibits cisplatin-induced apoptosis [297]. Sinomenine (**108**), an alkaloid isolated from an oriental medicinal herb *Sinomenium acutum,* which has been used to treat inflammatory diseases including rheumatism and arthritis [298]. Sinomenine (**108**) pretreatment was able to induce HO-1 expression in donor livers in a dose dependent manner and it protected the liver graft from cold ischemia/reperfusion injury [299]. Higenamine (**109**) is a bioactive alkaloid in *Aconitum carmichaeli,* which has been used as a heart stimulant and anti-inflammatory agent in traditional oriental medicine [300]. Higenamine (**109**) promotes M2 macrophage activation and reduces Hmgb1 expression dependent on HO-1 induction and then promotes locomotor function after spinal cord injury [301]. Camptothecin (**110**) is a potent anticancer alkaloid isolated from *Camptotheca acuminate* [302] as a strong inhibitor of the DNA-replicating enzyme topoisomerase I [303]. Jayasooriya et al. suggested camptothecin (**110**) also inhibits the invasion of cancer cells accompanied by suppression of MMP-9 and VEGF production by suppressing the PI3K/Akt-mediated NF-κB pathway and enhancing the Nrf2-dependent HO-1 pathway [304]. Berberine (**111**) is an isoquinoline alkaloid from *Coptis chinensis* with pharmacological effects such as hypoglycemic, antioxidant and anti-inflammatory activity [305,306,307]. Berberine (**111**) can protect against methotrexate-induced liver injury from oxidative stress and apoptosis, possibly through upregulating the Nrf2/HO-1 pathway and PPARγ [308]. Capsaicin (**112**) is a unique alkaloid that provides spicy flavor of the fruit of the genus *Capsicum* (peppers) [309]. Joung et al. found that capsaicin (**112**) induced expression of HO-1 that resulted in a transient increase in the phosphorylation of Akt and subsequently nuclear translocation of Nrf2, enhancing its binding to ARE [310]. Kim et al. suggested the anti-inflammatory activity of capsaicin (**112**) and another derivative, dihydrocapsaicin (**113**) is exerted through NO production and iNOS expression and induction of HO-1 [311]. Matrine (**114**) is a quinolizidine alkaloid isolated from *S. flavescentis*, and possesses antioxidant, anti-inflammatory and antitumor activity [312,313,314]. Matrine (**114**) may alleviate early brain injury after experimental subarachnoid hemorrhage in rats possibly via PI3K/Akt-mediated NF-κB inhibition and Keap1/Nrf2-dependent HO-1 induction [315]. Cordycepin, a bioactive adenosine derivative, which was found in *Cordyceps militarisa* known as a rare Chinese caterpillar fungus, has beneficial activity to circulatory, immune, respiratory and glandular systems [316]. Cordycepin exhibited protective effects on *N*-nitrosodiethylamine-induced hepatocellular carcinomas via the PI3K/Akt/mTOR and Nrf2/HO-1/NF-κB pathway in mice [317]. Chabamide (**115**) is a dimeric piperine initially discovered from *Piper chaba* [318]. Ngo et al. isolated a series of alkaloid with inhibitory activity on LPS-induced NO production in RAW264.7 from *Piper nigrum* (black pepper) [319]. Among the alkaloids, chabamide (**115**) especially inhibited LPS-induced NO production in bone marrow-derived macrophages, via inducing HO-1 expression at the transcriptional level and inducing nuclear translocation of Nrf2 [319]. Piperlongumine (**116**), an alkaloid from *Piper longum* (long pepper) was found to induce apoptosis of human breast cancer MCF-7 cells mediated by upregulation of HO-1 expression [320]. Lu et al. reported that HO-1 induction of piperlongumine (**116**) may also result in the inhibitory effect on Zika virus replication [321]. Six isosteroid alkaloids isolated from *Fritillaria cirrhosa* bulbus, a Chinese folk herb with antitussive, expectorant, antiasthma and anti-inflammatory properties demonstrated to protect murine RAW264.7 macrophages against cigarette smoke-induced oxidative stress [322]. They were cevanine or jervine type alkaloids and decreased the generation of ROS and increased the level of GSH via Nrf2 nuclear translocation and HO-1 expression via activating Nrf2 signaling pathway [322]. Peiminine (**117**, also known as verticinone) has long been studied as a major bioactive component of anti-inflammatory Chinese medicinal herbs *Fritillaria* sp. [323]. Luo et al. reported peiminine (**117**) ameliorates murine osteroarthritis anti-inflammatory activity induced by inhibition of Akt phosphorylation, the nuclear transfer of NF-κB and activated Nrf2/HO-1 signaling pathways [324]. Structures of the alkaloids and nitrogen-containing natural products (**107**–**117**) are presented in Figure 20.

## 10. Conclusions

Global life expectancy has been increased in modern times, but healthy life expectancy has not been so, as most people suffer from chronic diseases, metabolic syndromes, degenerative brain diseases or cardiovascular diseases in their old age. However, a number of investigations suggest a healthy lifestyle including moderate exercise, a balanced diet and avoiding stress may improve health, delay the aging process, prevent chronic diseases and eventually increase healthy life expectancy. Adequate intake of antioxidant supplements may synergistically work to prevent those diseases as the diseases are mostly resulted from the accumulation of oxidative stress [325]. HO-1 plays a pivotal role in the antioxidant and anti-inflammatory system in humans, and it is possibly modulated by a variety of natural products in edible sources as we discussed so far. The natural products from edible sources might be promising sources of safe and effective HO-1 inducing agents that help our body protect from the chronic diseases.
